# The effect of a theory of planned behavior-based education model on self-management and metabolic control in pregnant women with gestational diabetes: a randomized controlled trial

**DOI:** 10.1186/s12884-026-09217-8

**Published:** 2026-06-01

**Authors:** Sinem Guven Dinmez, Kafiye Eroglu

**Affiliations:** 1 Midwifery Department, Mudanya University, Bursa, Türkiye; 2 Nursing Department, Istanbul Atlas University, İstanbul, Türkiye

**Keywords:** Gestational diabetes, Diabetes education, Theory of planned behavior, Lifestyle modification, Motivational interviewing techniques, Nursing, Midwifery

## Abstract

**Background:**

Gestational diabetes mellitus (GDM) is a common pregnancy complication associated with adverse maternal and neonatal outcomes. Lifestyle modification is central to GDM management; however, optimal strategies for supporting self-management remain unclear. This study evaluated the effect of a personalized Theory of Planned Behavior (TPB)–based online education model supported by motivational interviewing (MI) on behavioral determinants and metabolic control in women with GDM.

**Methods:**

This randomized controlled trial was conducted in a public maternity hospital in Istanbul, Türkiye, with 66 pregnant women with GDM (intervention: *n* = 33; control: *n* = 33). Data were collected using the Gestational Diabetes Intention, Attitude, and Behavior Questionnaire (GDIAB-Q), a sociodemographic and clinical form, and medical records. Co-primary outcomes were changes in five GDIAB-Q subscales (attitude, subjective norm, perceived behavioral control, intention, and planning). Secondary outcomes included metabolic parameters (postprandial blood glucose, HbA1c, lipid profile, blood pressure, and body mass index). The intervention group received three TPB-based online sessions with MI during gestational weeks 28–30; controls received usual care. Effect sizes (Cohen’s d) and 95% confidence intervals were calculated. The study was registered at ClinicalTrials.gov (NCT04874922).

**Results:**

The predefined theory-informed success criterion for the co-primary outcome set was met. Significant between-group improvements were observed in attitude (d = 0.71, 95% CI: 0.21–1.21, *p* = 0.005), subjective norm (d = 0.80, 95% CI: 0.30–1.30, *p* = 0.002), and intention (d = 0.56, 95% CI: 0.06–1.05, *p* = 0.028). No significant differences were found for perceived behavioral control or planning. Secondary outcomes favored the intervention, with lower postprandial blood glucose (d = 0.51, *p* = 0.045), HbA1c (d = 0.50, *p* = 0.045), and BMI (d = 0.59, *p* = 0.021).

**Conclusion:**

This TPB-based online education model strengthened key behavioral determinants of self-management and was associated with early improvements in selected metabolic outcomes among women with GDM. Theory-driven, personalized education may support GDM self-management; longer-term follow-up is needed to assess sustained effects.

**Supplementary Information:**

The online version contains supplementary material available at 10.1186/s12884-026-09217-8.

## Relevance to clinical practice

The TPB-based online education model demonstrated significant contributions to GDM self-management and certain metabolic control variables. Therefore, nurses are encouraged to take an active role in the management of gestational diabetes, guide pregnant women in improving their self-management, and provide online consultation support without time or location constraints.

## Introduction

The prevalence of GDM, one of the most common complications during pregnancy, has been increasing globally due to the rising rates of obesity and sedentary lifestyles [1]. It is reported that over 21 million births worldwide are affected by maternal diabetes [2]. According to the International Diabetes Federation (IDF) (2019), 84% of pregnant women affected by hyperglycemia are diagnosed with GDM [3]. GDM is associated with a range of complications, from birth traumas to an increased risk of developing diabetes later in life, posing risks to maternal, fetal, and neonatal health. Given the significant short- and long-term health and financial burdens it creates, controlling GDM is of critical importance [1, 4].

Prior to initiating medical treatment, the primary approach to GDM management during the perinatal period is a combination of lifestyle modifications, including nutrition education, physical activity, and blood glucose monitoring [4, 5]. While lifestyle modification interventions alone can achieve glycemic control in 70–85% of women with GDM, individual differences should not be overlooked [4]. These individual differences, both modifiable (environmental factors, lifestyle habits) and non-modifiable (genetic predisposition, advanced maternal age, prior related conditions), are key variables in assessing risk factors and enabling early intervention [6, 7].

The literature suggests that nurses/midwives should evaluate each patient’s knowledge of diabetes, identify barriers to diabetes self-management, such as psychosocial needs, and tailor care plans individually [8]. Providing basic information on lifestyle changes is insufficient; instead, behavioral treatment strategies that support individuals’ self-care skills are emphasized [9, 10]. Nurses, who are closely connected with families and communities, play a crucial role [11]. They are known for their ability to deliver effective, high-quality care at lower costs and are highlighted as having a pivotal role in diabetes education. Additionally, they are positioned to facilitate behavior change through motivational and theoretically grounded programs, especially in evolving healthcare systems [12, 13].

Human behavior is a clear and observable response that occurs under specific conditions and is directed toward a particular goal. Thus, if the intention related to the goal is identified and the beliefs influencing intention development are understood, barriers to behavior can be removed, and motivation for the behavior can be enhanced [14]. In diabetes management for pregnant women, the most common areas requiring support are nutrition education, physical activity, and self-monitoring of blood glucose. Studies show that lifestyle change efforts typically target these areas [15]. It is reported that lifestyle interventions should be theory-driven and that theory-based interventions yield more successful outcomes [16]. One of the most frequently tested theories used to identify health-related behaviors is the TPB. This theory helps identify behaviors individuals find challenging to perform, providing advantages in motivation and individual planning. As such, it is considered a good choice for studies related to diabetes self-management [17].

The National Institute for Health and Care Excellence (NICE) recommends structured education for individuals with diabetes immediately after diagnosis [18]. After diagnosis (after the 24th week of pregnancy), nurses can provide approximately four months of education and counseling to women with GDM. This limited time frame can create social inequalities for some, such as time, transportation, and cost issues. These inequalities can be reduced through web-based learning programs and educational applications, which have been reported to be as effective as face-to-face education [19, 20]. Studies evaluating the effectiveness of face-to-face and remote education models led by nurses found no difference in behavior change. In qualitative analyses of participants who did not show behavior change, motivational issues were reported regardless of the method used [21]. Today, there are still debates about how interventions should be designed and how to account for individual differences and motivation. These insights suggest a need for a diabetes education structure that minimizes social disadvantages, prioritizes individuality and enhances motivation. This study aims to examine the effect of a TPB-based education model, designed to address barriers to diabetes management, on GDM self-management and metabolic control.

### Research questions

#### Primary research question

Does an online educational intervention based on the TPB improve pregnant women’s attitude, subjective norm, perceived behavioral control, planning, and intention (the subscales of the GDIAB-Q)?

#### Secondary research question

Are there significant between-group differences in metabolic control parameters (postprandial blood glucose, HbA1c, body mass index, lipid profile, and blood pressure) between pregnant women who received the TPB-based online education and those who received usual care?

## Methods

### Study design

The objectives, outcome variables, and analytical approach of this randomized controlled trial were pre-specified. All analyses were conducted according to the pre-defined protocol, which was prospectively registered in the ClinicalTrials.gov database (Identifier: NCT04874922, registered on 27 November 2021). The reporting of this study adheres to the CONSORT (Consolidated Standards of Reporting Trials) guidelines.

### Study setting

The study was conducted between May and November 2021 in the antenatal and perinatology clinics of a 648-bed tertiary obstetrics and gynecology training and research hospital located in Istanbul, Türkiye. In this hospital, all pregnant women at 24 gestational weeks routinely undergo an Oral Glucose Tolerance Test (OGTT), and follow-up care is provided in the respective clinics. Pregnant women diagnosed with GDM are referred to a diabetes education nurse for counseling. As part of the standard care, they receive a single face-to-face diabetes education session lasting approximately 30 min at the time of diagnosis, supported by visual materials and delivered by the hospital’s diabetes education nurse. No structured follow-up education is routinely provided beyond standard clinic visits. Women who require insulin therapy are subsequently followed up in the endocrinology clinic.

### Study participants

In Türkiye, GDM screening tests are routinely performed between the 24th and 28th gestational weeks; therefore, pregnant women within this gestational age range were included in the study.

For the sample size calculation, data from the third follow-up of the Self-Efficacy Scale in the reference study [22] were examined. Among its subdimensions, the largest difference was observed in the “Behavior Completion” subscale (Cohen’s *d* ≈ 0.73). This value corresponds to a moderate-to-large effect size (*d* ≈ 0.7) as reported in similar studies and was adopted as the expected effect size in the present study. The sample size for the primary outcome variable (GDIAB-Q constructs) was calculated a priori using G*Power (version 3.x). A two-tailed independent samples t-test model was applied, with the effect size defined as Cohen’s d. Based on assumptions of α = 0.05 and power (1–β) = 0.80, a total of 58 participants (29 in each group) were required.

**Fig. 1:**
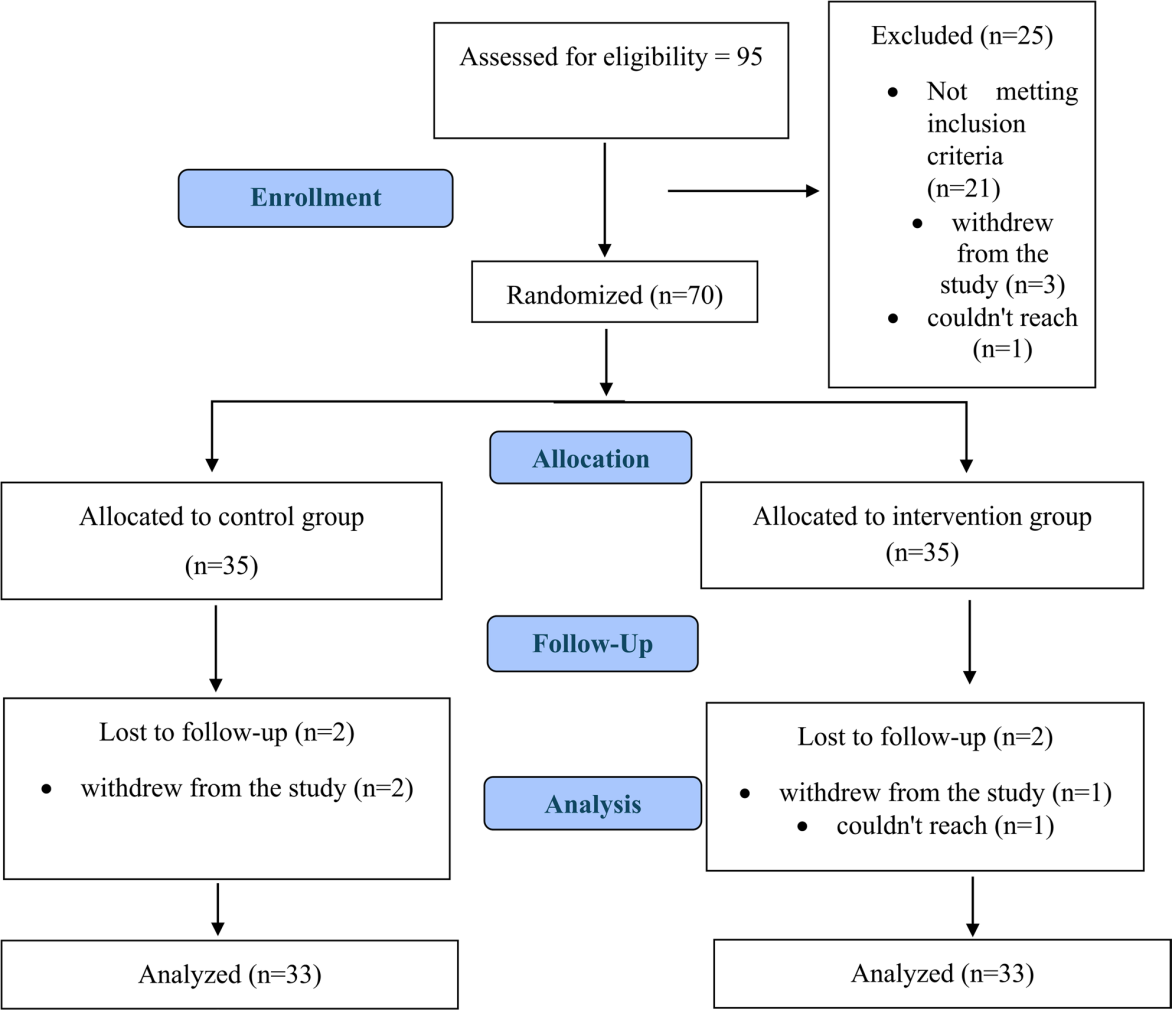
CONSORT flow diagram

Considering that small pilot samples may overestimate the effect size, the final calculation was based on literature-derived d values. Allowing for an anticipated 15–20% attrition rate, a total of 70 pregnant women (35 in the intervention and 35 in the control group) were initially recruited. The study was completed with 66 participants (33 per group) due to protocol deviations such as loss to follow-up or inability to contact (Fig. 1).

#### Inclusion criteria

Being in the 24th-28th week of pregnancy, having a first-time diagnosis of gestational diabetes, ability to communicate verbally, literacy, and access to a personal mobile phone and internet.

#### Exclusion criteria

Having a known systemic disease (such as hypertension, lipid disorders, high cholesterol, and endocrine diseases), a prior history of diabetes, multiple pregnancies, known fetal anomalies, known mental health issues, or the use of antidiabetic medications.

### Data collection tools

#### Descriptive data collection form

This form consists of 26 questions and was administered at the start of the study. It aimed to evaluate the sociodemographic and obstetric characteristics of the pregnant women, as well as features related to GDM (such as receiving diabetes education, influences on behaviors related to gestational diabetes, chronic illness, and medication use, family history of diabetes, nutrition, diet, and exercise).

#### MCV data collection form

This form was used to monitor the blood values of the pregnant women, including fasting blood glucose, postprandial blood glucose (1st-hour postprandial glucose), HbA1c, triglycerides, total cholesterol, HDL (high-density lipoprotein) cholesterol, Body Mass Index (BMI), and blood pressure. MCV data were measured at four points: between the 24th and 28th weeks of pregnancy (baseline), at the 32nd week (first measurement), at the 36th week (second measurement), and between the 37th-40th weeks (third measurement). All laboratory analyses were conducted at the hospital where the data were collected, and the results were obtained from the pregnant women’s digital health records.

#### The Gestational Diabetes Intention, Attitude, and Behavior Questionnaire (GDIAB-Q)

The GDIAB-Q is the Turkish version of the questionnaire based on the TPB, consisting of 14 items and 5 sub-dimension. It was used to assess the intentions, attitudes, and behaviors of pregnant women with GDM (Cronbach’s alpha (α): 0.88, item-total score correlation values: (0.354 − 0.713) [23, 24]. The questionnaire has three sections focused on behaviors related to physical activity, nutrition education, and weight control in pregnant women with gestational diabetes. Each section evaluates the sub-dimensions of attitude, subjective norm, perceived behavioral control, intention, and planning, all of which influence behavior. Items are scored from 1 to 7, with a maximum score of 100, and higher scores indicate stronger intentions. In other words, the higher the score in the sub-dimensions, the higher the diabetes self-management of the pregnant women is considered to be. The questionnaire was administered face-to-face at the beginning of the study (pre-test) and through online questions (via WhatsApp video call) at the end of the study (post-test).

### Data collection procedure

Data were obtained from 66 pregnant women in an antenatal and perinatology clinic between May and November 2021. Participants were randomized after completion of baseline assessments and prior to receipt of standard care education. A computer-generated randomization sequence was used, and group assignments were concealed until participants were enrolled. The clinic physicians and the diabetes education nurse were informed about the study. All data were collected by the researcher through regular visits to the clinic two to three times a week. Pregnant women who volunteered to participate were informed about the study, and written informed consent was obtained. This study was not participant-blinded due to the nature of the behavioral intervention. However, data analysts were blinded to group allocation during statistical analysis to reduce bias. Data collection was carried out following the randomization table to ensure equal group allocation. Participants were assessed in the order in which they were enrolled in the study to maintain consistency in the data collection process.

### Development of the TPB-based online education model for gestational diabetes

The change theory used in this study is based on the hypothesis that identifying pregnant women’s intentions toward lifestyle changes will lead to improved health outcomes through self-management behaviors. The TPB-based online education model was developed in accordance with updated, evidence-based international diabetes management guidelines [1, 3–5]. TPB was chosen because diabetes is a disease that can be effectively managed through behavior change.

The theory consists of three main components: Attitude, Subjective Norm, and Perceived Behavioral Control. Attitude refers to whether the pregnant women evaluate self-management behavior related to diabetes as positive or negative. Subjective Norm refers to the social companions (such as partner, mother, and friends) who influence what the pregnant women believe they should do regarding the target behavior. Perceived Behavioral Control represents how easy or difficult pregnant women perceive it is to perform self-management behavior related to diabetes. Underlying these components are the beliefs that influence these behaviors. The education aims to identify the beliefs influencing pregnant women in diabetes-related behavior and provide education, counseling, and motivational support. This approach creates a tailored educational model focused on the needs of pregnant women. The GDIAB-Q pretest data were used to identify participants’ beliefs, and the question “Who are the people influencing your behaviors related to gestational diabetes?” was used to determine social supporters (subjective norms). The social supporters identified by the pregnant women were invited to participate in the online sessions, particularly in the modules where resistance to change was observed (those with lower GDIAB-Q pretest scores). Each social supporter attended at least one session and provided verbal encouragement to the participants to maintain behavior change at the end of the session. The increase observed in the subjective norm component of the GDIAB-Q posttest scores was interpreted as an indication that the involvement of social supporters enhanced the participants’ motivation for diabetes self-management.

The education was planned in three modules: physical activity, nutrition education, self-monitoring, and diabetes control. Each module lasted approximately 45–60 min. The Gestational Diabetes Education Guide for Women, prepared by the researcher based on the literature, was used as the educational material. The question-and-answer and MI techniques were employed as the educational method. The goal of MI is to elicit the motivation needed for individuals to make behavioral changes. These techniques were employed exclusively when the pregnant women exhibited resistance to change during the educational process. The theoretical framework and the planned education intervention for lifestyle changes are presented in the table (Table 1). Prior to structuring the education model, the researcher participated in the training program “Examples of Online Diabetes Education During the Covid-19 Pandemic” and the certification program for “Motivational Interviewing Techniques.” Feedback was obtained from seven experts holding PhDs in the TPB, Motivational Interviewing Techniques, and GDM to evaluate the content validity of the education program and materials. The final version of the education program was shaped based on the recommendations provided by these experts.

**Table 1:** Details of lifestyle interventions and strategies to address constructs in theoretical frameworks

Education Intervention	Module 1: Medical Nutrition Therapy		Module 2: Physical Activity	Module 3: Self-Monitoring and Control of Blood Glucose
Objective:	Adaptation to Medical Nutrition Therapy (MNT)		Adaptation to Physical Activity Behavior (PA)	Enabling pregnant women to monitor and control their blood glucose (SMBG)
Time/Duration:	28th gestational week/45–60 min		29th gestational week / 45–60 min	30th gestational week/45–60 min
Content:	Basic information about MNT, importance of MNT in diabetes, meal planning, relationship between nutrition and blood glucose, effects of MNT on maternal and fetal health		Basic information about PA, the importance of PA in diabetes, proper activity planning and timing, the relationship between PA and blood glucose, the relationship between PA and nutrition	Principles of SMBG, blood glucose measurement skills, the importance of record-keeping, monitoring, and control, parameters used in glycemic control (HbA1c, blood glucose, blood pressure, lipids, and BMI)
Educational Material:	Guide for Women with Gestational Diabetes (GDM)		Guide for Women with GDM	Guide for Women with GDM
Teaching Technique:	Online consultation and Motivational Interviewing (MI) techniques where needed (based on pre-test scores from the TPB-based GDIAB-Q), using open-ended questions, reflective listening, summarizing, and giving effective advice		Online consultation and Motivational Interviewing (MI) techniques where needed (based on pre-test scores from the TPB-based GDIAB-Q), using open-ended questions, reflective listening, summarizing, and giving effective advice	Online consultation and Motivational Interviewing (MI) techniques where needed (based on pre-test scores from the TPB-based GDIAB-Q), using open-ended questions, reflective listening, summarizing, and giving effective advice
**Strategies to Address Constructs in the Theory of Planned Behavior (TPB)**				
Attitude (Behavioral Beliefs)	Identifying the pregnant woman’s positive and negative attitudes toward MNT in diabetes management	Identifying the pregnant woman’s positive and negative attitudes toward PA in diabetes management		Identifying the pregnant woman’s positive and negative attitudes toward SMBG and control in diabetes management
Subjective Norm (Normative Beliefs)	Identifying the social and professional supporters influencing the pregnant woman’s adherence to diet and inviting them to support MNT in the education	Identifying the social and professional supporters influencing the pregnant woman’s PA behavior and inviting them to support PA in the education		Identifying the social and professional supporters influencing the pregnant woman’s SMBG and control behaviors and inviting them to support SMBG in the education
Perceived Behavioral Control (Control Beliefs)	Determining the pregnant woman’s perceptions and past habits related to diabetes diet	Determining the pregnant woman’s perceptions and past habits related to PA		Determining the pregnant woman’s perceptions related to SMBG and control

### Intervention

This educational model was implemented as an intervention in three modules delivered individually and online (via WhatsApp Web) once a week during the 28th, 29th, and 30th weeks of pregnancy. The social supporters identified by the pregnant women (such as their mothers, spouses, or friends) were invited to participate particularly in the modules where resistance to change was observed (e.g., nutrition education). The researcher documented the participation and verbal contributions of social supporters in session notes to monitor the implementation process. The topics of each module were centered on diabetes self-management (nutrition education, physical activity, and self-monitoring and control of blood glucose), with priority given to the area where the pregnant women had the lowest pre-test scores on the GDIAB-Q. These low scores also indicated which aspects of diabetes self-management posed the greatest challenges for the women. Therefore, Motivational Interviewing (MI) techniques were applied specifically to areas where behavioral resistance was evident.

In each module, the Gestational Diabetes Education Guide for Women was used as the educational material. The guide included content such as the definition of diabetes, diabetes management (nutrition education, physical activity, monitoring of blood glucose and metabolic parameters, medical check-ups), the effects of diabetes on maternal and fetal health, and the continuity of metabolic control during the birth and postpartum periods. Visual aids were also included. MI techniques were utilized at points where resistance to behavior change was encountered. According to MI, the readiness of the pregnant women for change, or their stage in the change process (contemplation, readiness, or action), was first assessed. A shared agenda was then created with the pregnant women to begin the education, and the initial control was given to them to ensure active participation. Within the routine clinical context of the study setting, women with diet-controlled gestational diabetes are supported through lifestyle education delivered by trained diabetes education nurses or midwives, in accordance with standard clinical practice. Referral to a dietitian or endocrinologist is reserved for cases requiring pharmacological treatment.

The educational sessions were conducted using approaches that addressed the specific needs of the pregnant women, such as asking open-ended questions, supporting their opinions, reflective listening, summarizing, and providing practical advice. At the end of each session, an action plan was created and recorded with the pregnant women to strengthen their commitment to change. Compliance with the action plan was assessed in the following session. Each session lasted between 45 and 60 min. After the 30th week of pregnancy, follow-up and counseling were continued with the intervention group through online and phone communication during the 32nd, 36th, and 36th to 40th weeks of pregnancy.

### Definition and evaluation of protocol adherence

Protocol adherence was defined as the completion of the planned intervention sessions, measurement time points, and data collection stages by the participants as outlined in the study protocol.

For the intervention group, adherence was evaluated based on the following criteria:

1. Participation in all three online education modules conducted between the 28th and 30th gestational weeks.
2. Planning of the subsequent session content individually at the end of each module and documentation of this plan by the researcher.
3. Sharing of medical data and participation in counseling sessions at baseline (24–28 weeks) and during follow-up periods (32nd, 36th, and 37–40th gestational weeks).

For the control group, adherence was defined as receiving standard hospital education and completing data collection at all scheduled measurement points (24–28, 32, 36, and 37–40 gestational weeks).

Attendance monitoring was conducted by the researcher through session attendance logs and WhatsApp communication records. Participants who attended all three sessions and provided complete measurement data were classified as protocol adherent. Failure to attend one or more sessions, inability to reach the participant, or missing follow-up data were considered indicators of protocol non-adherence.

### Study protocol

#### Intervention group

Pregnant women with GDM who met the inclusion criteria completed the GDIAB-Q (pre-test), the Descriptive Data Collection Form, and the MCV data collection form at the start of the study (between the 24th and 28th weeks of pregnancy). The participants in this group received the TPB-Based Education Model for GDM online (via WhatsApp Web) once a week for three sessions during the 28th, 29th, and 30th weeks of pregnancy. MCV data were collected online and by phone during the 32nd, 36th, and 36th-40th weeks of pregnancy. The GDIAB-Q (post-test) was administered between the 36th and 40th weeks of pregnancy. Participants were provided a phone number for further support, and counseling was offered until delivery.

#### Control group

The purpose of the study was explained to pregnant women with GDM who met the inclusion criteria. At the start of the study (between the 24th and 28th weeks of pregnancy), participants completed the GDIAB-Q (pre-test), the Data Collection Form, and the MCV data collection form. MCV data were collected online and by phone during the 32nd, 36th, and 36th-40th weeks of pregnancy. The GDIAB-Q (post-test) was administered between the 36th and 40th weeks of pregnancy. Pregnant women in the control group received standard care from the hospital’s diabetes education nurse. Standard care consisted of a single face-to-face education session lasting approximately 30 min at the time of diagnosis, delivered by the hospital diabetes education nurse. In addition to verbal counseling, the control group received the standard printed educational material routinely provided by the hospital at the time of diagnosis. This session covered general information about gestational diabetes and basic lifestyle recommendations. No structured follow-up education or individualized counseling was provided beyond routine clinic visits.

### Measurable outcomes

#### Co-primary outcomes

Co-primary outcomes were defined as changes in the five TPB constructs measured by the GDIAB-Q (attitude, subjective norm, perceived behavioral control, intention, and planning) from baseline to post-test. Based on the TPB framework, a theory-informed success criterion was specified a priori. Intervention effectiveness was considered supported if (1) a significant improvement was observed in intention and (2) at least one of its theoretical determinants (attitude, subjective norm, or perceived behavioral control) also demonstrated significant improvement compared with the control group.

#### Secondary outcomes

Secondary outcomes included metabolic control variables (FPG, PPBG, HbA1c, lipid profile, blood pressure, and BMI).

#### Statistical methods

The sample size calculation was performed using MedCalc Statistical Software version 12.7.7. Data were analyzed using IBM SPSS version 23 (IBM Corporation, Armonk, New York, USA). This study was designed as a superiority randomized controlled trial. The primary causal contrast was defined as the between-group difference in change scores from baseline to post-intervention for the co-primary outcome set. Analyses were conducted using a per-protocol approach, including participants who completed the intervention and post-test assessments. Normality of data distribution was assessed using the Shapiro–Wilk and Kolmogorov–Smirnov tests. Independent Samples t-tests were used for normally distributed variables, and Mann–Whitney U tests were applied for non-normally distributed variables.

One-Way Analysis of Variance and the Duncan Test were conducted for multiple comparisons in comparing customarily distributed data in groups of three or more. The Kruskal-Wallis Test was used to compare the data that were not generally distributed in the triple groups, and the Dunn Test was used for multiple comparisons. Chi-square, Yates Correction, and Fisher’s Exact tests were used to compare categorical data, Wilcoxon Test was used for comparisons based on two times within the group, and Friedman Test was used for comparisons made according to three or more times. To reduce the probability of Type I error in multiple comparisons, the Dunn test with Bonferroni correction was applied. Cohen’s d was used to assess the magnitude of differences between groups and for repeated measurements at two times, and Kendall’s W was used to assess the strength of change in repeated measurements (3 or more).

In analyzing the relationship between the variables, the data that did not comply with the normal distribution was evaluated with The Spearman Rho Rank Correlation Coefficient. Results were presented as frequency (percentage) for categorical variables, mean ± standard deviation, and median (minimum-maximum) for quantitative variables. Significance was evaluated at the *p* < 0.05 level.

## Results

### Descriptive characteristics

Of the 70 participants initially enrolled, 66 (94.3%) completed the intervention and post-test assessments and were included in the per-protocol analysis, while four participants were excluded due to protocol non-adherence. This high adherence rate indicates that the study procedures and the online education model were well accepted and feasible for pregnant women with GDM. The descriptive characteristics of the pregnant women in both groups are similar (age, education, weight, employment status, income status, smoking). It was determined that almost half of both groups had at least two births and had their last birth with a normal vaginal delivery and had no obstetric problems during pregnancy, and the average birth weight of their previous babies was between 3000 and 4000 g.

It was determined that almost all of the pregnant women had not received any training on GDM before, did not have any chronic diseases, did not use regular medication and did not have any first-degree relatives with diabetes. The pregnant women reported that the three individuals who most influenced their diabetes-related behaviors (such as nutrition, physical activity, and blood glucose monitoring) were, in order of frequency, their husbands (53%), mothers (28%), and healthcare professionals (19%). Most of the pregnant women reported that they consume all food groups (carbohydrate, protein, fat) daily and that they have not yet applied a nutrition diet, and that they walk 1–2 h per week as physical activity. No statistically significant differences were found between groups in descriptive, obstetric, and gestational diabetes-related characteristics (*p* > 0.05), indicating that the groups were comparable at baseline (Table 2).

**Table 2:** Social-demographic and obstetric characteristics of the total samples and the comparison between groups at baseline (*N* = 66)

Characteristics	Intervention group (*n* = 33) *n* (%) or mean ± SD	Control group (*n* = 33) *n* (%) or mean ± SD	*p* value
Age	27.81 ± 9.4	27.00 ± 9.3	0.746*
BMI	27.21 ± 2.85	28.82 ± 2.97	0.204*
Normal	18 54.5	11 33.3	
Overweight	13 39.4	20 60.6	
Obesity	2 6.1	2 6.1	
Educational level			0.817*
Primary school or below	12 36.4	12 36.4	
Secondary school	12 36.4	14 42.4	
College	9 27.3	7 21.2	
Occupational status			0.763**
Employed	8 24.2	6 18.2	
Unemployed	25 75.8	27 81.8	
Gravidity			0.628*
1	12 36.4	9 27.3	
2	6 18.2	8 24.2	
3	11 33.3	14 42.4	
4 >	4 12.1	2 6.1	
Parity			0.205*
1	5 26.3	11 45.8	
2	10 52.6	11 45.8	
3	4 21.1	1 4.2	
4 >	0 0.0	1 4.2	
Last delivery status			0.929**
Cesarean delivery	7 35.0	7 29.2	
Vaginal delivery	13 65.0	17 70.8	
Birth weight of last baby			0.734**
3000 g/ -	1 5.0	2 8.3	
3000–4000 g	16 80.0	20 83.3	
4000 g / +	3 15.0	2 8.3	

*Pearson Chi-square

**Yates Correction

### Results of the GDIAB-Q

The co-primary outcome set comprised the five TPB constructs measured by the GDIAB-Q. According to the predefined theory-informed success criterion, intervention effectiveness required significant improvement in intention together with improvement in at least one of its theoretical determinants (attitude, subjective norm, or perceived behavioral control). Between-group comparisons showed that the intervention group had significantly higher posttest mean scores in attitude, subjective norm, and intention compared with the control group. Specifically, attitude scores (*p* = 0.005) demonstrated a moderate-to-large effect (95% CI: 0.21–1.21), subjective norm scores (*p* = 0.002) showed a large effect (95% CI: 0.30–1.30), and intention scores (*p* = 0.028) indicated a moderate effect (95% CI: 0.06–1.05) (Table 3). The mean posttest scores in the intervention group were 94.4 (range: 66–100) for attitude, 94.4 (range: 66–100) for subjective norm, and 88.9 (range: 50–100) for intention. No significant between-group differences were observed for perceived behavioral control or planning (*p* > 0.05). Within-group comparisons revealed statistically significant improvements across all GDIAB-Q subdimensions (attitude, subjective norm, perceived behavioral control, planning, and intention). In the intervention group, all subdimensions showed highly significant changes (*p* < 0.001), with large effect sizes (Cohen’s d = 3.61, 3.64, 3.58, 3.70, and 3.75, respectively). Although significant within-group differences were also observed in the control group (*p* < 0.05), corresponding effect sizes were small to moderate (Table 3). The very large within-group effect sizes likely reflect pre–post standardization within a short time interval and should be interpreted cautiously.

**Table 3:** Findings of the pregnants on the gestational diabetes İntention, attitude and behavior questionnaire scores

Sub-Dimensions		Intervention (n=33)		Control (n=33)		Analysis	p*	*Cohen’s d (%95 CI)*
		Mean ± SD	Median (min-max)	Mean ± SD	Median (min-max)			
Attitude	Pre-test	71.38 ± 13.26	72.22 (50 – 94.44)	65.99 ± 9.6	66.67 (44.44 - 88.89)	418	0.101	0.408 (-0.08 : 0.89)
	Post-test	92.59 ± 8.52	94.44 (66.67 - 100)	85.86 ± 11.29	88.89 (55.56 - 100)	332	0.005	0.712 (0.21 : 1.21)
Analysis		-5.026		-4.883				
p**		<0.001		<0.001				
*Cohen’s d (%95 CI)*		3.613 (2.48 : 4.73)		3.227 (2.16 :4.27)				
Subjective Norm	Pre-test	68.52 ± 10.36	72.22 (44.44 - 83.33)	62.79 ± 12.15	61.11 (33.33 - 83.33)	395.5	0.053	0.484 (-0.01 :0.97)
	Post-test	92.09 ± 8.57	94.44 (66.67 - 100)	84.01 ± 11.35	83.33 (55.56 - 100)	308.5	0.002	0.803 (0.3 : 1.3)
Analysis		-5.036		-5.029				
p**		<0.001		<0.001				
*Cohen’s d (%95 CI)*		3.644 (2.52 : 4.79)		3.622 (2.52 : 4.79)				
Perceived Behavioral Control	Pre-test	73.48 ± 13.25	75 (50 - 91.67)	69.19 ± 12.92	66.67 (41.67 - 91.67)	438.5	0.167	0.339 (-0.15 : 0.82)
	Post-test	93.18 ± 8.7	91.67 (58.33 - 100)	90.91 ± 10.49	91.67 (66.67 - 100)	492.5	0.477	0.165 (-0.32 : 0.65)
Analysis		-5.03		-5.039				
p**		<0.001		<0.001				
*Cohen’s d (%95 CI)*		3.626 (2.49 : 4.74)		3.654 (2.51 : 4.78)				
Intention	Pre-test	62.46 ± 12.73	66.67 (27.78 - 77.78)	58.75 ± 12.27	61.11 (27.78 - 77.78)	440.5	0.178	0.333 (-0.15 : 0.82)
	Post-test	87.04 ± 11.68	88.89 (50 – 100)	79.29 ± 14.72	83.33 (50 – 100)	374.5	0.028	0.557 (0.06 : 1.05)
Analysis		-5.07		-4.967				
p**		<0.001		<0.001				
*Cohen’s d (%95 CI)*		3.754 (2.59 : 4.9)		3.442 (2.34 : 4.52)				
Planning	Pre-test	53.7 ± 12	55.56 (27.78 - 72.22)	55.05 ± 12.29	55.56 (22.22 - 77.78)	519.5	0.745	0.079 (-0.4 : 0.56)
	Post-test	80.47 ± 11.96	83.33 (50 - 100)	77.1 ± 12.33	77.78 (44.44 - 94.44)	452.5	0.232	0.294 (-0.19 : 0.78)
Analysis		-5.016		-4.964				
p**		<0.001		<0.001				
*Cohen’s d (%95 CI)*		3.583 (2.45 : 4.69)		3.434 (2.33 : 4.51)				

*Mann-Whitney U Test, **Wilcoxon Test, CI Confidence Interval

*p*-value‡: Probability Value

### Results of the MCV

Secondary outcome analyses showed significant between-group differences in selected metabolic indicators (PPBG, HbA1c, and BMI). The mean PPBG levels were significantly lower in the intervention group compared with the control group (*p* = 0.045), with a medium effect size (95% CI: 0.02–1.00). Similarly, HbA1c levels were significantly lower in the intervention group (*p* = 0.045), also indicating a medium effect size (95% CI: 0.02–1.00). In addition, BMI values were significantly lower in the intervention group (*p* = 0.021), with a medium effect size (95% CI: 0.10–1.08) (Table 4). No significant between-group differences were observed in fasting blood glucose, triglycerides, total cholesterol, HDL cholesterol, or systolic/diastolic blood pressure values (*p* > 0.05). Examination of participants’ insulin use showed that three women in the control group required insulin therapy (one between weeks 32–36 and two between weeks 36–40 of pregnancy), whereas only one woman in the intervention group initiated insulin treatment between weeks 28–30. Due to the small number of participants requiring insulin, these data were not included in the statistical analysis.

**Table 4:** Comparison of metabolic control variables of pregnant women between and within groups

			Intervention (n=33)				Control (n=33)		Analysis	p*	*Cohen’s d (%95 CI)*
			Mean ± SD		Median (min-max)		Mean ± SD	Median (min-max)			
FBG^1^(mg/dL)		Beginning	91.82 ± 11.62		89 (76 - 124)		95.42 ± 15.69	89 (76 - 129)^b^	507	0.630	0.119 (-0.36 :0.6)
		1st measurement	96.97 ± 10.97		96 (76 - 124)		95.94 ± 10.64	92 (79 - 122)^ab^	511	0.662	0.106 (-0.32 : 0.65)
		2nd measurement	97.09 ± 10.84		96 (79 - 124)		99.00 ± 12.37	96 (84 - 142)^a^	512	0.676	0.103 (-0.38 : 0.59)
		3rd measurement	95.39 ± 10.09		96 (77 - 114)		95.18 ± 10.75	93 (78 - 121)^ab^	572	0.724	0.087 (-0.4 : 0.57)
		Mean FBG	95.32 ± 9.45		94 (79 - 116)		96.39 ± 11.2	93 (82 - 125)	543	0.980	0.005 (-0.48 : 0.49)
		Analysis	8.042				10.274				
		p**	0.051				0.016				
		*Kendall’s W (%95 CI)*	0.081 (-0.186 : 0.348)				0.104 (-0.16 : 0.368)				
PPBG^2^(mg/dL)		Beginning	149.97 ± 18.48		148 (108 - 194)		158.45 ± 22.16	151 (139 - 230)^b^	444	0.197	0.321 (-0.17 : 0.81)
		1st measurement	151.94 ± 12.93		150 (128 - 179)		167.64 ± 25.26	163 (138 - 249)^ab^	336	0.007	0.697 (0.2 : 1.19)
		2nd measurement	154.61 ± 18.01		156 (119 - 205)		172.06 ± 32.67	166 (138 - 279)^a^	380	0.035	0.538 (0.04 : 1.03)
		3rd measurement	156.76 ± 17.08		155 (119 - 188)		167.39 ± 25.68	167 (138 - 236)^ab^	430	0.142	0.368 (-0.12 : 0.85)
		Mean PPBG	153.32 ± 13.70		155 (124 - 191)		166.39 ± 25.45	158 (140 - 243)	388	0.045	0.51 (0.02 : 1.00)
		Analysis	3.881				14.823				
		p**	0.275				0.002				
		*Kendall’s W (%95 CI)*	0.039 (0.234 : 0.312)				0.15 (-0.107 : 0.407)				
HbA1c^3^(%)		Beginning	6.34 ± 0.57		6.3 (5.7 - 8.9)^b^		6.61 ± 0.6	6.5 (5.4 - 8.6)	346	0.011	0.66 (0.16 : 1.15)
		1st measurement	6.32 ± 0.53		6.3 (5.5 - 8.6)^b^		6.62 ± 0.53	6.5 (5.8 - 8.3)	334	0.007	0.705 (0.2 : 1.2)
		2nd measurement	6.42 ± 0.55		6.4 (5.6 - 8.3)^ab^		6.67 ± 0.5	6.6 (5.9 - 7.8)	384	0.039	0.524 (0.524 : 1.01)
		3rd measurement	6.62 ± 0.59		6.5 (5.7 - 8.1)^a^		6.58 ± 0.42	6.5 (6 - 7.5)	543.5	0.990	0.003 (-0.48 : 0.49)
		Mean HbA1c^3^	6.43 ± 0.49		6.4 (6 - 8)		6.62 ± 0.48	6.6 (6 - 8)	388.5	0.045	0.508 (0.02 : 1.00)
		Analysis	12.35				3.832				
		p**	0.006				0.28				
		*Kendall’s W (%95 CI)*	0.125 (-0.136 : 0.385)				0.039 (-0.234 : 0.312)				
Triglycerides(mg/dL)		Beginning	123.39 ± 38.30		108 (78 - 196)^b^		128.36 ± 28.98	136 (69 - 172)^b^	488	0.469	0.179 (-0.31 : 0.66)
		1st measurement	129.67 ± 35.53		124 (79 - 202)^b^		135.45 ± 32.52	144 (80 - 198)^b^	482	0.423	0.198 (-0.29 : 0.68)
		2nd measurement	157.21 ± 51.34		145 (92 - 299)^a^		147.70 ± 39.38	149 (81 - 209)^a^	492	0.497	0.166 (0.16 : 1.15)
		3rd measurement	153.45 ± 55.25		146 (12 - 278)^a^		160.61 ± 47.82	168 (79 - 248)^a^	505	0.612	0.125 (-0.36 : 0.61)
		Mean Triglyc.	140.93 ± 42.93		127 (91 - 232)		143.03 ± 36.09	149 (80 - 205)	509	0.649	0.112 (-0.37 : 0.59)
		Analysis	65.751				55.077				
		p**	<0.001				<0.001				
		*Kendall’s W (%95 CI)*	0.664 (0.503 : 0.836)				0.556 (0.371 : 0.742)				
Total Cholesterol(mg/dL)	Beginning		168.79 ± 53.03	166 (100 - 267)^cd^		176.12 ± 55.92		172 (99 - 299)^b^	509	0.644	0.112 (-0.37 : 0.59)
	1st measurement		172.27 ± 48.33	168 (100 - 255)^bc^		179.15 ± 54.18		172 (98 - 276)^b^	507	0.626	0.119 (-0.36 : 0.6)
	2nd measurement		179.94 ± 54.56	168 (99 - 268)^a^		190.85 ± 61.3		176 (100 - 290)^a^	489	0.477	0.176 (-0.31 : 0.66)
	3rd measurement		179.82 ± 49.58	168 (103 - 255)^ad^		201.79 ± 70.12		178 (98 - 324)^a^	452	0.233	0.295 (-0.19 : 0.78)
	Mean T. Choles.		175.2 ± 50.58	162.3 (101 - 257)		186.98 ± 59.43		174 (101 - 290)	478	0.390	0.211 (-0.27 : 0.69)
	Analysis		18.184			41.245					
	p**		<0.001			<0.001					
	*Kendall’s W (%95 CI)*		0.184 (-0.068 : 0.435)			0.417 (0.204 : 0.629)					
HDL^4^(mg/dL)	Beginning		57.94 ± 8.19	58 (45 - 79)^c^		55.73 ± 6.39		56 (46 - 71)^b^	622	0.319	0.247 (-0.24 : 0.73)
	1st measurement		58.48 ± 8.14	57 (44 - 81)^bc^		60.45 ± 7.26		60 (48 - 71)^b^	438	0.169	0.341 (-0.15 : 0.83)
	2nd measurement		60.45 ± 8.6	59 (48 - 82)^ab^		62.82 ± 7.43		63 (49 - 75)^a^	433	0.150	0.358 (-0.13 : 0.84)
	3rd measurement		62.48 ± 8.36	62 (49 - 79)^a^		65.21 ± 6.97		67 (51 - 77)^a^	427	0.131	0.378 (-0.11 : 0.86)
	Mean HDL		59.84 ± 7.88	59 (47 - 80)		61.05 ± 5.39		62 (50 - 73)	450	0.223	0.302 (-0.18 :0.79)
	Analysis		33.79			34.83					
	p**		<0.001			<0.001					
	*Kendall’s W (%95 CI)*		0.341 (0.115 : 0.567)			0.342 (0.116 : 0.568)					
Blood Pressure(Systolic)(mm/Hg)	Beginning		119.7 ± 10.15	120 (90 - 140)		120.00 ± 10		120 (100 - 150)	527	0.810	0.055 (-0.43 : 0.54)
	1st measurement		120.3 ± 9.18	120 (100 - 140)		120.91 ± 9.8		120 (100 - 140)	534	0.887	0.033 (-0.45 : 0.52)
	2nd measurement		120.3 ± 8.83	120 (100 - 140)		121.82 ± 11.85		120 (110 - 160)	533	0.876	0.036 (-0.45 : 0.52)
	3rd measurement		122.73 ± 10.69	120 (110 - 150)		125.45 ± 11.48		130 (100 - 160)	440	0.158	0.335 (-0.15 : 0.82)
	Mean Systolic		120.76 ± 8.56	120 (100 - 138)		122.05 ± 8.94		120 (105 - 145)	518	0.727	0.084 (-0.4 : 0.57)
	Analysis		3.545			8.832					
	p**		0.315			0.051					
	*Kendall’s W (%95 CI)*		0.06 (-0.238 : 0.309)			0.089 (-0.177 : 0.355)					
Blood Pressure(Diastolic)(mm/Hg)	Beginning		78.18 ± 9.5	80 (60 - 100)		75.45 ± 9.71		70 (60 - 100)	457	0.238	0.279 (-0.21 : 0.76)
	1st measurement		77.58 ± 9.69	80 (60 - 100)		75.76 ± 8.67		80 (60 - 90)	496	0.511	0.154 (-0.33 : 0.64)
	2nd measurement		78.18 ± 8.46	80 (60 - 90)		77.88 ± 9.27		80 (60 - 100)	532	0.865	0.039 (-0.44 : 0.52)
	3rd measurement		80.61 ± 8.99	80 (70 - 110)		78.48 ± 9.72		80 (60 - 110)	477	0.353	0.214 (-0.27 : 0.7)
	Mean Diastolic		78.64 ± 8.39	80 (63 - 95)		76.89 ± 8.29		75 (60 - 93)	487	0.457	0.182 (-0.3 : 0.66)
	Analysis		7.279			9.000					
	p**		0.064			0.051					
			0.074 (-0.195 : 0.342)			0.091 (-0.175 : 0.357)					
BMI^5^(kg/m^2^)	Beginning		27.21 ± 2.85	27 (21.3 - 34.4)^c^		28.82 ± 2.97		30 (23.7 - 35.5)^c^	360	0.018	0.609 (0.11 :1.1)
	1st measurement		27.35 ± 2.87	27 (21.6 - 34.8)^c^		29.05 ± 2.95		29 (24 - 35.9)^c^	354	0.014	0.631 (0.13 : 1.12)
	2nd measurement		28.08 ± 2.87	28 (22.3 - 35.6)^b^		29.65 ± 3.02		29 (24.4 - 36.3)^b^	372	0.026	0.566 (0.07 : 1.06)
	3rd measurement		30.51 ± 3.01	30 (25 - 38.3)^a^		32.13 ± 3.17		33 (26.1 - 38.5)^a^	377	0.032	0.548 (0.05 : 1.04)
	Mean BMI		28.29 ± 2.88	28 (23 - 36)		29.91 ± 2.99		30 (25 - 37)	365	0.021	0.591 (0.1 : 1.08)
	Analysis		96.553			95.499					
	p**		<0.001			<0.001					
	*Kendall’s W (%95 CI)*		0.975 (0.931 : 1.019)			0.975 (0.930 : 1.019)					

a-d: There is no difference between groups with the same letter

*CI* Confidence Interval

*Mann Whitney U Test, **Friedman Test, Kendall’s W Test

^1^Fasting Blood Glucose, ^2^ Postprandial Blood Glucose, ^3^ Glycolyzed Hemoglobin, ^4^High-Density Lipoprotein, ^5^ Body Mass Index

## Discussion

The predefined theory-informed success criterion for the co-primary outcome set was met, as significant improvements were observed in intention together with its theoretical determinants, particularly attitude and subjective norm. These findings support the proposed TPB pathway, whereby changes in cognitive and normative beliefs contribute to strengthened behavioral intention [14, 25]. Lifestyle modification is a cornerstone of gestational diabetes management; however, pregnant women often require structured support to sustain motivation and integrate recommended behaviors into daily routines [4–6]. In this randomized controlled trial, the TPB-based online education model was associated with meaningful improvements in key behavioral determinants of self-management, with additional beneficial effects observed in selected secondary metabolic outcomes.

The intervention group demonstrated significant between-group improvements in attitude, subjective norm, and intention, whereas perceived behavioral control and planning did not differ significantly between groups. Although both groups showed within-group improvements over time, effect sizes were notably larger in the intervention group, suggesting that the observed behavioral changes were largely attributable to the educational model.

Following the intervention, pregnant women’s attitudes toward nutrition, physical activity, and weight management improved with a moderate-to-large effect size. This aligns with previous research indicating that positive attitudes facilitate healthy behavioral intentions and that individualized education enhances engagement in self-care behaviors [26, 27]. Similarly, subjective norm improved substantially when social supporters identified by participants (e.g., spouses, mothers, and healthcare professionals) were actively involved in the intervention. This finding reinforces existing evidence that social support plays a critical role in motivating diabetes self-management [26, 28, 29].

Although perceived behavioral control did not differ significantly between groups, scores increased over time in both groups and approached the upper limits of the scale, suggesting high perceived competence. This may reflect a ceiling effect or the influence of routine clinical care. Moreover, TPB distinguishes perceived from actual behavioral control [25, 30]; structural constraints such as time limitations, physical discomfort, and family responsibilities may restrict behavioral implementation despite strong intentions. Future qualitative studies could further elucidate contextual barriers influencing actual control.

The intervention also resulted in a significant improvement in intention, with a moderate effect size, highlighting clinically meaningful enhancement of motivation. Consistent with TPB theory, intention is shaped by attitude and subjective norm and represents a central driver of sustained health behavior [25, 31, 32]. However, the translation of intention into action may depend on available resources and ongoing contextual support.

Planning did not differ significantly between groups, possibly due to high baseline levels, although a positive trend was observed in the intervention group. Planning behaviors may require longer follow-up periods to demonstrate measurable change and are often strengthened when outcomes are perceived as personally meaningful [33–35]. The incorporation of motivational interviewing within the educational sessions may have supported early motivational processes in this sample, even if measurable planning behaviors had not yet fully emerged, consistent with prior research on motivational interviewing [36–38].

Regarding secondary metabolic outcomes, significant improvements in postprandial blood glucose, HbA1c, and BMI were observed in the intervention group, suggesting favorable effects on glycemic control and weight management. These findings are consistent with previous nurse-led and empowerment-based diabetes education programs [39–42]. The absence of differences in some metabolic parameters may be attributed to the relatively short intervention duration, as physiological adaptations often lag behind behavioral changes. Weight management remains central to glycemic regulation, and the observed reductions in BMI suggest clinically relevant benefits [43–47]. Behavioral improvements may precede metabolic change, which typically evolves more gradually over time [6, 39, 41, 48]. It should also be considered that outcome measurements were conducted within a relatively short gestational window (between approximately 28 and 36–40 weeks of pregnancy). Therefore, the magnitude of metabolic change observed in this study reflects short-term adaptations rather than long-term glycemic regulation. The limited duration between baseline and follow-up assessments may partly explain why some metabolic parameters did not demonstrate significant between-group differences.

Overall, the present findings indicate that TPB-based education can strengthen key behavioral determinants of self-management and contribute to early metabolic improvements. However, behavioral and physiological changes appear to progress at different rates, underscoring the importance of sustained support and longer-term follow-up in future studies.

### Strengths ands limitations

This study is among the first to integrate a Theory of Planned Behavior (TPB)-based online education model into the management of gestational diabetes mellitus. A key strength of the study is the randomized controlled design and the clear definition of a co-primary outcome set grounded in a theory-informed framework. In Türkiye, women with diet-controlled gestational diabetes are routinely referred to diabetes education nurses for lifestyle-based diabetes self-management education, in accordance with standard clinical practice. Referral to a dietitian or endocrinologist is reserved for cases requiring pharmacological treatment or more complex metabolic management. Therefore, intervention effects should be interpreted within the context of this local care pathway. The use of effect size estimates and confidence intervals enhances the interpretability and clinical relevance of the findings. In addition, the high protocol adherence rate supports the feasibility and acceptability of the intervention among pregnant women with GDM.

However, several limitations should be considered. Recruitment was conducted during the COVID-19 pandemic, which limited the achievable sample size. In relatively small samples, random baseline imbalances may occur despite proper randomization. In this study, baseline differences were observed in BMI and HbA1c between groups, and no baseline-adjusted analyses were performed; therefore, metabolic findings should be interpreted with caution. First, analyses were conducted using a per-protocol approach, which may overestimate intervention effects compared with an intention-to-treat analysis. Second, although a co-primary outcome framework was applied, multiple comparisons across behavioral constructs may increase the risk of type I error. Third, the intervention group received more structured and prolonged educational contact compared with usual care, and therefore some effects may be attributable to differences in intensity rather than solely to the TPB-based framework. Fourth, the relatively short follow-up period may have limited the detection of changes in certain behavioral constructs (e.g., planning) and in some metabolic parameters, as physiological adaptations often require longer timeframes. Finally, the intervention was appropriate for women whose GDM could be managed primarily through lifestyle modification; thus, findings cannot be generalized to women requiring pharmacological treatment.

## Conclusion

According to the findings of this randomized controlled trial, the online education model based on the TPB had statistically and clinically significant effects on the behavioral antecedents of GDM management attitude, subjective norm, and intention. The effect sizes observed in these subdimensions (Cohen’s *d* = 0.6–0.8) were within the moderate to large range. The intervention strengthened key behavioral determinants of diabetes self-management, including attitude, subjective norm, and intention, and supported motivational processes underlying behavior change.

Beyond behavioral improvements, notable outcomes were also observed in metabolic control indicators. Significant reductions were found in postprandial blood glucose, HbA1c, and body mass index (BMI) in the intervention group, with a medium effect size observed for BMI (Cohen’s *d* = 0.591). This finding suggests that the online educational intervention was associated with early improvements in selected metabolic indicators. As one of the first examples of integrating a TPB-based online educational model into GDM management, this study highlights the potential of individualized, accessible, and motivation-enhancing digital education interventions. The findings demonstrate that such models can be effectively utilized in nursing and midwifery practices to support behavioral change among women with gestational diabetes.

### Recommendations

Nurses and midwives should take an active role in GDM management by planning individualized counseling processes that consider pregnant women’s personal characteristics, sociocultural factors, and educational needs.

Online education programs grounded in theoretical frameworks such as TPB and incorporating Motivational Interviewing (MI) techniques may be considered for integration into clinical practice to enhance self-management and treatment adherence. These digital interventions should be expanded given their accessibility across time and place and their potential to support sustainable behavioral change.

Future research should evaluate the applicability of this TPB-based model in diverse socioeconomic and cultural populations. Long-term follow-up studies are also needed to investigate the persistence of the model’s effects, its sustained impact on metabolic control, and its relationship with birth outcomes.

## Supplementary Information

Supplementary Material 1.

## Data Availability

The data supporting the findings of this study are available from the corresponding author upon reasonable request.

## Abbreviations

GDIAB-Q: Gestational Diabetes Intention
GDM: Gestational Diabetes Mellitus
MCV: Metabolic Control Variables
MI: Motivational Interviewing
TPB: Theory of Planned Behavior
FBG: Fasting Blood Glucose
PPBG: Postprandial Blood Glucose
HbA1c: Glycolyzed Hemoglobin
HDL: High-Density Lipoprotein
BMI: Body Mass Index

## Declarations

The ethics committee of the study was approved by Zeynep Kamil Training and Research Hospital Clinical Research Ethics Committee (ethics committee approval, numbered 128, dated December 18, 2019; additional approval dated December 23, 2020, and numbered 2012KAEK-71-130) and the study permission of Kanuni Sultan Süleyman Training and Research (April 27, 2021, and E-15916306-604.01.01-3514). Written informed consent was obtained from the participants by giving them an enlightening text under the principles of the Declaration of Helsinki. No payment was made to the participants or the institution.
