# Supplementary material for: The effect of a theory of planned behavior-based education model on self-management and metabolic control in pregnant women with gestational diabetes: a randomized controlled trial
Source: BMC Pregnancy Childbirth. 2026 Jun 1;26:844. doi: 10.1186/s12884-026-09217-8 (PMC13445864; doi:10.1186/s12884-026-09217-8)
Supplement: Supplementary file 1 [file 12884_2026_9217_MOESM1_ESM.pdf]

Training Guide for Women with Gestational Diabetes Based on the Theory of Planned

PhD. Sinem DİNMEZ
Prof. Kafiye EROĞLU

Table of Contents

1. **Introduction to the Theory Used in the Preparation of the Guide**
   1.1. Theory of Planned Behavior
2. **Introduction to the Interview Technique Used in the Training**
   2.1. Motivational Interviewing Techniques
3. **General Information About the Guide**
   3.1. Purpose of the Guide
   3.2. Target Group
   3.3. General Objectives
   3.4. Content and Use of the Training Guide
4. **Session 1:** Medical Nutrition Therapy Based on the Theory of Planned Behavior
5. **Session 2:** Physical Activity Based on the Theory of Planned Behavior
6. **Session 3:** Monitoring and Control of Diabetes Based on the Theory of Planned Behavior

1. Introduction to the Theory Used in the Preparation of the Guide

1.1. Theory of Planned Behavior (TPB)

The Theory of Planned Behavior (TPB) is one of the most widely used social-psychological theories explaining human behavior. Developed by Icek Ajzen in 1985, the theory posits that intention is the most important determinant for performing a goal-oriented behavior, and that intention itself is influenced by motivational factors.

The theory consists of three main components:

Attitude: The individual's evaluation of a behavior as positive or negative. The development of attitudes is based on **behavioral beliefs**.

Subjective Norm: The perceived social pressure from significant others about whether the individual should or should not perform the behavior. It is influenced by **normative beliefs**, referring to the beliefs of people in one's social environment.

Perceived Behavioral Control: The individual's perception of how easy or difficult it is to perform the behavior. It is shaped by **control beliefs**, which are based on past experiences and perceived barriers or facilitators.

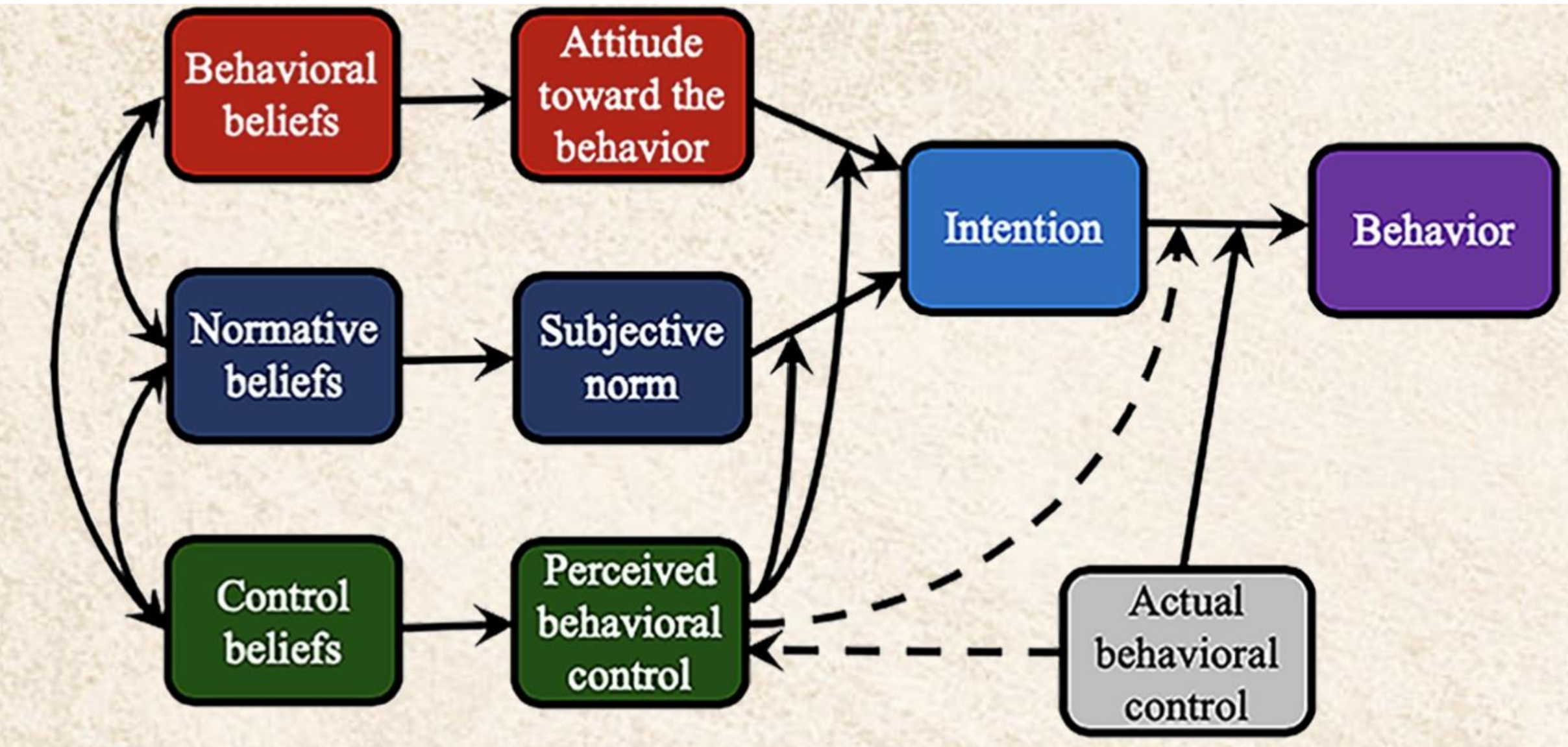

Figure 1. Planned Behaviour Model (https://people.umass.edu/aizen/tpb.diag.html#null-link)

According to Ajzen, if the factors influencing these beliefs can be modified, the behavior itself can also be influenced. Therefore, by eliminating the barriers that prevent a behavior from

occurring, the desired behavior can be achieved (Ajzen, 1991; Ajzen & Driver, 1992; Bosnjak, Ajzen, & Schmidt, 2020).

Since interventions for the management of Gestational Diabetes Mellitus (GDM) directly target behavior change, individuals must adopt and implement these changes. The key component for performing a behavior is the **behavioral intention**, which encompasses motivational factors such as past experiences, social support, and education. Through the planned educational intervention, it is aimed to motivate pregnant women to adhere to medical nutrition therapy, engage in physical activity, and carry out diabetes monitoring and control behaviors. To ensure the effectiveness of the training, the implementation is designed to employ **Motivational Interviewing Techniques (MI)**.

2. Introduction to the Interview Technique Used in the Training

2.1. Motivational Interviewing Techniques

Developed by Miller and Rollnick, Motivational Interviewing (MI) is a directive and client-centered approach used to elicit behavioral change by helping individuals resolve ambivalent feelings. The primary goal of MI is to stimulate the individual's intrinsic motivation for change and to ensure active participation in the change process. This approach is particularly effective for individuals who are reluctant or ambivalent about making changes (Rollnick & Miller, 1995).

The four fundamental components of motivational interviewing are: engaging, focusing, evoking, and planning. Throughout these stages, interactive techniques are used, including open-ended questioning, affirmations, reflective listening, summarizing, offering advice upon request, and emphasizing personal responsibility.

In each stage of behavior change, MI begins by assessing **importance** and **confidence** scales to promote motivation for change. These methods will be used in the educational program. When participants demonstrate resistance to change, appropriate resistance-handling strategies of MI will be applied, such as reflective listening, shifting focus, emphasizing personal choice, exploring future perspectives, and walking alongside the participant (Ögel, 2009).

The training consists of three stages — medical nutrition therapy, physical activity, and diabetes control/monitoring — structured according to the components of the Theory of Planned

Behavior. The MI techniques will be applied throughout the sessions. There is no hierarchy among the three sessions; participants will decide the sequence, emphasizing autonomy.

3. General Information About the Training Guide

3.1. Purpose of the Guide

The purpose of this training guide is to assist educators in promoting behavioral change among pregnant women with GDM by addressing factors influencing adherence to medical nutrition therapy, physical activity, and diabetes monitoring and control.

3.2. Target Group

The target group consists of pregnant women between 24–28 weeks of gestation who have been diagnosed with GDM, have no other systemic disease, have no prior history of GDM, are literate, have internet access, and plan to give birth at the hospital where the study is conducted.

3.3. General Objectives of the Training Guide

By the end of the training, participants will be able to:

- Explain that GDM is a manageable health condition.
- Recognize the importance of diabetes management, including medical nutrition therapy, physical activity, and diabetes monitoring/control.
- Apply medical nutrition therapy as part of diabetes management.
- Engage in regular physical activity.
- Monitor blood glucose levels regularly.
- Attend regular medical follow-ups.
- Explain the effects of diabetes on maternal and infant health.
- Record metabolic control parameters (HbA1C, triglycerides, total cholesterol, HDL, fasting and postprandial blood glucose, and blood pressure) within target ranges.
- Maintain body mass index (BMI) within the recommended range.
- Prevent maternal complications (e.g., preterm delivery, hypertension, hyperglycemia/hypoglycemia, prolonged hospitalization).

- Prevent neonatal complications (e.g., macrosomia, dystocia, fetal acidosis, respiratory distress syndrome, birth trauma, neonatal hypoglycemia, hyperbilirubinemia).
- Attend postpartum medical check-ups within the first 12 weeks to assess the risk of Type 2 diabetes.

3.4. Content and Application of the Training Guide

The training program, developed to support effective self-management of GDM, consists of three sections. After an overview of the training, each key component of diabetes management (medical nutrition therapy, physical activity, diabetes monitoring/control) is addressed within the TPB framework.

The training is delivered in three weekly sessions:

1. **Session 1:** Medical Nutrition Therapy
2. **Session 2:** Physical Activity
3. **Session 3:** Diabetes Monitoring and Control

There is no priority among the topics; participants can start with the one they prefer. Each session is designed around the three main components of TPB — **Attitude**, **Subjective Norm**, and **Perceived Behavioral Control** — which correspond to behavioral, normative, and control beliefs respectively. Based on participants' responses to the **Diabetes Intention, Attitude, and Behavior Scale (DIABS)**, individual differences will be considered to tailor the emphasis on specific components.

During education related to normative beliefs, each participant's previously identified **social supporter** (as noted in the personal data collection form) will be invited to the relevant session. The implementation phase of the training will utilize **Motivational Interviewing (MI)** techniques.

The content of the training aligns with the national guidelines of the Ministry of Health and the Provincial Health Directorate for the management of gestational diabetes (İstanbul İl Sağlık Müdürlüğü, 2020; Türkiye Halk Sağlığı Kurumu, 2015). Evaluation of educational outcomes will be conducted using the DIABS, Metabolic Control Variables Form, and Maternal and Neonatal Outcome Assessment Forms.

4.Session 1: Medical Nutrition Therapy Based on the Theory of Planned Behavior

Topic: Medical Nutrition Therapy in Gestational Diabetes

Duration: 60 minutes

Main Objective: To enable pregnant women to adhere to medical nutrition therapy by addressing their behavioral, normative, and control beliefs influencing gestational diabetes management.

Sub-Objectives

- Strengthen positive perceptions (benefits, motivations) and modify negative perceptions (barriers, disadvantages) related to behavioral beliefs about medical nutrition therapy.
- Identify and engage social supporters (normative beliefs) to enhance adherence behaviors through collaboration.
- Address and modify control beliefs that hinder adherence to dietary recommendations, fostering confidence and positive beliefs toward maintaining the diet.

Learning Objectives

Behavioral Beliefs:

- Explain the role of medical nutrition therapy in diabetes management.
- Describe the relationship between medical nutrition therapy and blood glucose control.
- Demonstrate appropriate dietary behaviors aligned with diabetes management.

Normative Beliefs:

- Identify individuals who can provide social support for adherence to dietary therapy.
- Collaborate effectively with supporters to sustain adherence.

Control Beliefs:

- Identify facilitating and hindering factors that influence adherence to dietary therapy.
- Develop strategies to overcome barriers and maintain adherence behaviors.

Training Method

The training will be delivered online through **interactive methods** such as open-ended questions, affirmations, reflective listening, summarizing, and advice-giving in accordance with **Motivational Interviewing (MI)** principles.

Educational Materials:

- Educational booklet for women with gestational diabetes
- Visual materials related to nutrition and diabetes management

Introduction (15 minutes)

Gestational diabetes can be effectively managed through behavioral interventions. The session will begin with **motivational interviewing techniques** to explore participants' feelings, concerns, and expectations about the training. After a brief introduction to the content and objectives, participants will choose which diabetes management topic to begin with, fostering motivation and autonomy.

Main Session (30 minutes)

After establishing the agenda, the session will focus on **medical nutrition therapy**. Participants' attitudes, beliefs, and motivations regarding dietary adherence will be explored. Ambivalence will be elicited to help participants recognize conflicting thoughts (e.g., wanting a healthy baby but struggling to give up sweets).

Through reflective discussion, participants will recognize discrepancies between their goals and behaviors. Motivation to change will emerge as ambivalence decreases. The participant's readiness to change will be assessed using the **Stages of Change Model**, which includes contemplation, preparation, action, maintenance, relapse, and precontemplation.

If the participant is not yet ready, resistance-handling strategies from MI will be employed — such as reflective listening, shifting focus, emphasizing personal choice, and exploring future goals.

Eliciting Change Talk

- **Open-ended questions:** "What are the advantages of following your diet plan?"
- **Importance scale:** "On a scale from 0 to 10, how important is it for you to keep your blood sugar within normal limits?"
- **Confidence scale:** "On a scale from 0 to 10, how confident are you that you can follow your diet plan?"
- **Decisional balance:** Exploring pros and cons of current versus desired behavior.

Example Dialogue:

"You mentioned that not following your diet allows you to eat what you want, but it also makes you feel unwell when your blood sugar rises. When you do follow your diet, you said you feel more energetic and your sugar levels are stable. It sounds like you value both freedom and health — what would make it easier for you to choose the healthier path more often?"

Planning and Social Support

At this stage, participants will set personal goals related to medical nutrition therapy. Their **social supporters** (identified earlier) will be invited to join. Questions such as *"What will be your first step toward maintaining your diet?"* will help the participant establish actionable goals.

If the participant requests detailed information, the educator will explain — using neutral, third-person language — the health implications of gestational diabetes and the importance of dietary adherence for both mother and baby.

Throughout the session, the four MI components — engaging, focusing, evoking, and planning — will guide the educator's approach, ensuring empathy, autonomy, and collaboration.

Evaluation (15 minutes)

The educator summarizes the discussion and plans established during the session. The participant is informed about digital follow-up options for any further questions or counseling needs.

5.Session 2: Physical Activity Based on the Theory of Planned Behavior

Topic: Promoting Regular Physical Activity Among Women with Gestational Diabetes

Duration: 45 minutes

Main Objective: To encourage pregnant women to engage in regular physical activity by addressing behavioral, normative, and control beliefs influencing diabetes management.

Sub-Objectives

- Strengthen positive perceptions (benefits, motivations) and modify negative perceptions (barriers) related to physical activity.
- Identify and collaborate with social supporters to sustain motivation and adherence to physical activity.
- Address and change control beliefs that prevent participation in physical activity, enhancing confidence and self-efficacy.

Learning Objectives

Behavioral Beliefs:

- Explain the importance of physical activity in diabetes management.
- Describe the relationship between physical activity and blood glucose control.
- Demonstrate the ability to engage in regular physical activity.

Normative Beliefs:

- Identify individuals who can provide social support for engaging in physical activity (social supporters).
- Maintain motivation for physical activity through collaboration with supporters.

Control Beliefs:

- Identify factors (barriers/facilitators) influencing engagement in physical activity.
- Strengthen beliefs about the ease and feasibility of regular physical activity.

Training Method

The training will be conducted online using **Motivational Interviewing (MI)** techniques — open-ended questions, affirmations, reflective listening, summarizing, and advice-giving.

Educational Materials:

- Educational booklet for women with gestational diabetes
- Visual aids related to exercise and diabetes management

Introduction (10 minutes)

The educator reviews goals and outcomes from the previous session on medical nutrition therapy. The discussion then shifts to physical activity, and the participant is asked which topic she prefers to begin with. This establishes the session's agenda and ensures active engagement.

Main Session (30 minutes)

Once the agenda is established, the focus turns to the participant's beliefs and attitudes toward physical activity. The educator facilitates discussion to uncover **ambivalence** — for example, a participant may wish to control her blood sugar but avoids exercise due to fatigue or discomfort.

As the conversation progresses, the participant recognizes discrepancies between her intentions and behaviors, which fosters motivation for change. Readiness for change is assessed using the **Stages of Change Model** (contemplation, preparation, action, maintenance, relapse, precontemplation). If the participant is not ready, MI resistance-handling techniques are applied: reflective listening, shifting focus, emphasizing autonomy, exploring future perspectives, and walking alongside.

Eliciting Change Talk

- **Open-ended question:** "What are the benefits of regular physical activity for you?"
- **Importance scale:** "On a scale from 0 to 10, how important is physical activity for you?"
- **Confidence scale:** "On a scale from 0 to 10, how confident are you that you can maintain regular activity?"
- **Decisional balance:** Exploring pros and cons of being active versus inactive.

Example Dialogue:

“You mentioned that avoiding physical activity helps you avoid fatigue, but it also causes your blood sugar to rise quickly. When you are active, you said you feel more energetic and can manage your sugar levels better. It sounds like staying active helps you feel in control — what would make it easier for you to keep this routine?”

Planning and Social Support

During the planning phase, the participant identifies personal goals and strategies for maintaining regular physical activity. Her **social supporter** (previously identified) is invited to join the session. The educator facilitates discussion by asking, *“What will be your first step toward including regular activity in your routine?”*

If requested, the educator explains in detail — using neutral, third-person language — the importance of physical activity in diabetes management. For instance:

“Regular physical activity such as walking, aerobic exercises, and prenatal pilates is known to help maintain healthy blood sugar levels and reduce pregnancy-related complications.”

Throughout the session, MI’s four processes — engaging, focusing, evoking, and planning — guide communication. Techniques such as open-ended questioning, affirmation, reflection, and summarizing are used flexibly according to participants’ needs.

Evaluation (5 minutes)

The educator summarizes the session’s discussions, including goals and planned activities. Participants are informed about the option for digital follow-up if they have questions or need additional support.

6.Session 3: Monitoring and Control of Diabetes Based on the Theory of Planned Behavior

Topic: Monitoring and Control of Diabetes (Blood Glucose Monitoring, Insulin Use, Medical Checkups, Birth, and Postpartum Care)

Duration: 45 minutes

Main Objective: To encourage pregnant women to regularly monitor and control diabetes by addressing their behavioral, normative, and control beliefs.

Sub-Objectives

- Strengthen positive beliefs and motivation related to regular monitoring and control behaviors.
- Identify and involve social supporters to promote adherence to monitoring routines.
- Address and modify control beliefs that hinder diabetes self-management, fostering confidence and positive attitudes.

Learning Objectives

Behavioral Beliefs:

- Explain the importance of monitoring and control (blood parameters, weight, blood pressure, and medical visits) in diabetes management.
- Regularly perform blood glucose monitoring, administer insulin if prescribed, and attend medical checkups as recommended.

Normative Beliefs:

- Identify individuals who provide support for monitoring and control behaviors.
- Strengthen motivation for regular monitoring with the help of social supporters.

Control Beliefs:

- Recognize facilitating and hindering factors affecting monitoring and control practices.
- Strengthen beliefs regarding the ease and importance of performing regular monitoring.

Training Method

The training is conducted online using **Motivational Interviewing (MI)** methods — open-ended questions, affirmations, reflective listening, summarizing, and collaborative advice.

Educational Materials:

- Educational booklet for women with gestational diabetes
- Visual materials on glucose monitoring, insulin use, and postpartum care

Introduction (10 minutes)

The educator reviews goals from the previous session on physical activity and assesses progress. Participants are informed that this is the final educational session, but digital or phone consultations will continue periodically for data collection and counseling. The topic of **monitoring and control** is introduced, and the session agenda is set collaboratively.

Main Session (30 minutes)

The educator facilitates discussion on participants' beliefs and experiences related to diabetes monitoring and control. Ambivalence is explored to uncover discrepancies between intentions and behaviors — for example, a participant may want her baby to be healthy but avoids regular blood glucose checks or medical appointments.

As ambivalence decreases, motivation for behavior change increases. Readiness to change is assessed using the **Stages of Change Model** (contemplation, preparation, action, maintenance, relapse, precontemplation). If participants are not yet ready, MI resistance-management techniques are applied, including reflective listening, shifting focus, emphasizing autonomy, and exploring future-oriented thinking.

Eliciting Change Talk

- **Open-ended question:** "What are the advantages of regular monitoring and control for you?"
- **Importance scale:** "On a scale from 0 to 10, how important is it for you to follow your monitoring routine?"
- **Confidence scale:** "On a scale from 0 to 10, how confident are you that you can manage your monitoring schedule?"
- **Decisional balance:** Exploring advantages and disadvantages of adherence versus non-adherence.

Example Dialogue:

"You mentioned that not monitoring your blood sugar helps you avoid finger pricks and time tracking, but it also prevents you from knowing how your sugar levels fluctuate. When you do monitor regularly, you said you feel more in control and can take preventive action. It seems like monitoring gives you peace of mind — what would help you make it a consistent habit?"

Planning and Social Support

The educator supports participants in creating personalized monitoring and control plans. Each participant's **social supporter** (previously identified) is invited to attend this stage. Together, they identify steps to achieve consistent monitoring (e.g., "What will be your first step to ensure you check your blood sugar regularly?").

If participants request detailed information, the educator provides evidence-based explanations in neutral, third-person language:

"Regular monitoring of blood glucose before and after meals is essential for maintaining target glucose levels and preventing complications. Consistent medical checkups ensure early detection of any health issues for both mother and baby."

Throughout the session, MI's four processes — engaging, focusing, evoking, and planning — guide interaction. Techniques such as open-ended questioning, reflection, and summarizing are used to maintain empathy and collaboration.

Evaluation (5 minutes)

The educator summarizes the discussion and goals established for monitoring and control. Participants are reminded that they can contact the educator digitally for additional support. Educational sessions are completed by the **30th week of gestation**, with follow-up data collection and counseling planned at **32, 36, and 40 weeks** via digital or phone communication.

Note: The training plan was developed according to the principles of individualized education program design (Kargın, 2007; Şenyuva & Taşocak, 2007).

References

Ajzen, I. (1991). The theory of planned behavior. Organizational Behavior and Human Decision Processes, 50(2), 179–211. https://doi.org/10.1016/0749-5978(91)90020-T

Ajzen, I., & Driver, B. (1992). Application of the Theory of Planned Behavior to leisure choice. Journal of Leisure Research, 24(3), 207–224. https://doi.org/10.1080/00222216.1992.11969889

Bosnjak, M., Ajzen, I., & Schmidt, P. (2020). The Theory of Planned Behavior: Selected recent advances and applications. Europe's Journal of Psychology, 16(3), 352–356. https://doi.org/10.5964/ejop.v16i3.3107

İstanbul İl Sağlık Müdürlüğü. (2020). Gestational Diabetes Booklet. İstanbul, Turkey.

Kargın, T. (2007). Educational evaluation and individualized education program development process. Ankara University Faculty of Educational Sciences Journal of Special Education, 8(1), 1–15.

Ögel, K. (2009). Motivational interviewing technique. Türkiye Klinikleri Journal of Psychiatry Special Topics, 2(2), 41–44.

Rollnick, S., & Miller, W. R. (1995). What is motivational interviewing? Behavioural and Cognitive Psychotherapy, 23(4), 325–334. https://doi.org/10.1017/S135246580001643X

Şenyuva, E., & Taşocak, G. (2007). Nurses' patient education activities and the patient education process. Florence Nightingale Journal of Nursing, 15(59), 100–106.

Türkiye Halk Sağlığı Kurumu. (2015). Educator's Guide for Adults with Diabetes. Ankara, Turkey.
